# Research progress in clinical trials of stem cell therapy for stroke and neurodegenerative diseases

**DOI:** 10.1002/ibra.12095

**Published:** 2023-03-05

**Authors:** Shan‐Shan Yan, Senio Campos de Souza, Zhen‐Dong Xie, Yong‐Xin Bao

**Affiliations:** ^1^ Department of Anesthesiology Southwest Medical University Luzhou China; ^2^ State Key Laboratory of Quality Research in Chinese Medicine, Institute of Chinese Medical Sciences University of Macau Macau SAR China; ^3^ Institute for Bioengineering of Catalonia University of Barcelona Carrer de Baldiri Reixac Barcelona Spain; ^4^ Qingdao Women and Children's Hospital Qingdao University Qingdao China

**Keywords:** Alzheimer's disease, cerebral hemorrhage, cerebral ischemia, Parkinson's disease, stem cell treatment

## Abstract

The incidence of stroke and neurodegenerative diseases is gradually increasing in modern society, but there is still no treatment that is effective enough. Stem cells are cells that can reproduce (self‐renew) and differentiate into the body, which have shown significance in basic research, while doctors have also taken them into clinical trials to determine their efficacy and safety. Existing clinical trials mainly include middle‐aged and elderly patients with stroke or Parkinson's disease (mostly 40–80 years old), mainly involving injection of mesenchymal stem cells and bone marrow mesenchymal stem cells through the veins and the putamen, with a dosage of mostly 10^6^–10^8^ cells. The neural and motor functions of the patients were restored after stem cell therapy, and the safety was found to be good during the follow‐up period of 3 months to 5 years. Here, we review all clinical trials and the latest advances in stroke, Alzheimer's disease, and Parkinson's disease, with the hope that stem cell therapy will be used in the clinic in the future to achieve effective treatment rates and benefit patients.

## Graphical abstract

Here, we review all clinical trials and the latest advances in stroke (cerebral ischemia and cerebral hemorrhage) and neurodegenerative diseases (Alzheimer's disease and Parkinson's disease), focused on cure rates, details, and so on. Also, we hope that stem cell therapy will be used in the clinic in the future to achieve effective treatment rates and benefit patients.

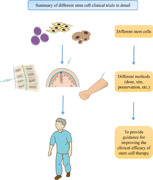

## 1 INTRODUCTION

Stem cells are cells with cell proliferation and potency, and have great potential for use in regenerative medicine. These cells have two properties, the ability to self‐renew by going through numerous cycles of cell growth and cell division, while maintaining the undifferentiated state and the capacity to differentiate into specialized cell types over the course of life. In terms of potency, there are five types currently under study: totipotent or omnipotent, pluripotent, multipotent, oligopotent, and unipotent cells.

The first cell type is totipotent stem cells. Totipotent stem cells are able to divide and differentiate into whole organisms, have the highest differentiation potential, and allow cells to form embryonic and extra‐embryonic structures.^1^ One example is a fertilized egg. After about 4 days, the inner cell mass of the blastocyst becomes pluripotent, and this structure is the source of pluripotent cells. The second cell type is pluripotent stem cells (PSCs). PSCs are cells that form all germ layers, but are not extra‐embryonic structures.^2^ An example of PSCs is embryonic stem cells (ESCs), which are derived from the inner cell mass of the preimplantation embryo. In 2006, researchers identified conditions that allow some mature adult cells to reprogram to an embryonic stem‐like state.^3^ These reprogrammed stem cells, called induced pluripotent stem cells (iPSCs), function similarly to PSC. Their emergence is of great interest for regenerative medicine now and in the future.^4^ The third cell type is multipotent stem cells. Multipotent stem cells have a narrower spectrum of differentiation than PSCs.^5^ However, they can specialize in discrete cells of specific cell lineages. This allows them to specialize in discrete cells of a particular cell lineage. A classic example is hematopoietic stem cells, which can differentiate into a variety of blood cells but not into cells outside the hematopoietic system. After differentiation, hematopoietic stem cells become oligopotent cells. The fourth cell type is oligopotent stem cells. Oligopotent stem cells can differentiate into several cell types.^6^ An example is bone marrow stem cells, which can divide into white blood cells but not into red blood cells. The fifth cell type is unipotent stem cells. Unipotent stem cells can only form one cell type because of their minimal differentiation ability and repeatable division, which makes them promising for therapeutic applications in regenerative medicine.^7^ These stem cells can remain quiescent (nondividing) for long periods until they are activated by the normal need for more cells to maintain and repair tissue. It can be obtained directly from the corresponding parts by the corresponding method of extraction, separation, and culture.

Clinical trials are studies conducted in humans that are monitored by government regulators to determine the extent to which a medical method can be used to treat a disease or injury compared with existing alternatives. The trials are structured to collect data on the safety and efficacy of new medical treatments. Similar to traditional drug trials, stem cell trials aim to find improved ways to prevent, treat, or diagnose diseases. At present, many clinical trials of stem cells are conducted every year, including hematological diseases, diabetes, liver cirrhosis, respiratory diseases, bone and joint diseases, and so on, showing great potential for clinical application. A study on half‐cycle and open‐label of human body transplant pancreatic endodermal cells from PSCs into VC—02 microtubes to directly vascularize cells, showed 63% of VC—02 implanted cells and expression of insulin at 3–12 months after surgery, suggested that PSCs can multiply to the required amount and differentiate into islet‐like tissues.^8^ In another clinical trial, umbilical cord mesenchymal stem cells (UCMSCs) combined with autologous bone marrow mononuclear cells (ABM‐MNC) stem cell transplantation (SCT) for type 1 diabetes were followed up for 8 years and it was found that this combination of stem cells reduced the incidence of chronic diabetic complications.^9^ More recently, researchers conducted a clinical trial in 219 patients using MSC infusions to improve decompensated cirrhosis, and significant improvement in liver function was observed during 48 weeks of follow‐up.^10^ In another study, human adipose‐derived mesenchymal stem cells were used to synthesize cartilage tissue and used in spinal orthopedic surgery in patients with osteoarthritis, and it was found that patients who received cartilage tissue and implants were in better condition than those who only underwent implantation surgery.^11^ At present, stem cell therapy for neurological diseases shows great potential. Recent studies found that magnetic nanovesicles derived from iron oxide nanoparticle (IONP)‐harboring MSC can drastically improve the ischemic‐lesion targeting and the therapeutic outcome.^12^ Neurons derived from hiNPC grafts received direct synaptic inputs from host neurons in patterns similar to corresponding endogenous neurons in the intact brain, suggesting that hiNPC contributes to functional recovery from stroke through the direct contribution of new neurons.^13^ Transplantation of BDNF‐modified human umbilical cord mesenchymal stem cell (hUC‐MSC)‐derived dopaminergic‐like neurons improved the apomorphine‐induced rotation behavior of Parkinson's disease (PD) rats.^14^ Here, we review the current clinical trials of stem cells in stroke (cerebral ischemia, cerebral hemorrhage) and neurodegenerative diseases (Alzheimer's disease [AD], PD), to introducing the progress of clinical trials, guiding the future direction of clinical research and clinical transformation, as well as finding improved methods for prevention, treatment, or diagnosis of diseases.

### 1.1 Literature search

The search situation of the four parts of the article is listed as follows, the source of literature retrieval is PubMed and the restriction of each article type was “clinical trials” (Table 1).

**Table 1:** Literature search and selection.

Keywords	Number of documents retrieved			The literature content corresponds to the table
	All	Recent 5 years	Finally included^a^	
“Intracerebral hemorrhage” and “stem cell”	17	6	4	Table 2
“Cerebral ischemia”, “Brain ischemia” and “stem cell”	57	19	29	Table 3
“Parkinsonian disorders”, “Parkinson disease”, and ”stem cell“	11	4	9	Table 4
“Alzheimer's disease” and “stem cell”	11	8	2	Table 5

^a^ Articles irrelevant to the corresponding subject and preclinical trials were screened out.

### 1.2 Stem cell clinical trials for stroke

#### 1.2.1 Stem cell treatment in intracerebral hemorrhage (ICH)

There were four valid articles related to stem cell treatment in ICH. The phase of clinical trials, cell interventions, dosage, infusion site, number of the trials (number of controls), main outcomes, safety, and year were extracted from the manuscript (Table 2).

**Table 2:** Summary of clinical trials of stem cells in the treatment of intracerebral hemorrhage (ICH).

Cells	Dosage	Site	Number of trials (controls)	Phase	Main outcomes	Safety	Year
Allogeneic MSCs	3 × 10^8^ (3)/6 × 10^8^(6)	Intracerebroventricular	9	I	Transplantation was well tolerated.	Safe	2018^15^
Autologous MSCs	<5 × 10^8^	MCA	10 (10)	/	Improved clinical outcomes.	Safe	2018^16^
Allogeneic OECs, NPCs, UCMSCs, SCs	1– 5 × 10^6^	Intracranial parenchyma, intrathecal, and venous	10	/	The neurological function was improved (during 6–24 months of follow‐up) (The Barthel index score and clinical neurological deficit scale score increased).	Safe	2013^17^
None	/	/	32		CD34(+) progenitor cells may be involved in the functional recovery of ICH patients.	/	2011^18^

*Note*: Phase refers to the phase of clinical trials. Dosage in the header refers to the cell dose. Cell doses were re‐calculated at 60 kg for each patient if the dose was only stated in terms of the number of cells per kilogram (cells/kg). The judgment criteria of security is the patients were well‐tolerated, and no serious adverse events occurred during follow‐up.

Abbreviations: ICH, intracerebral hemorrhage; MCA, middle cerebral artery; MSCs, mesenchymal stem cells; NPCs, neural progenitor cells; OECs, olfactory ensheathing cells; SCs, Schwann cells; UCMSCs, umbilical cord mesenchymal stromal cells.

Stem cell therapy for ICH includes MSCs, olfactory ensheathing cells, neural progenitor cells, umbilical cord mesenchymal cells, and Schwann cells. The doses used vary from 1 × 10^6^ to 5 × 10^8^ injections, and the injection sites are concentrated in the brain (intraventricular, MCA), intrathecally, and venous. The scales used for evaluation included the Barthel index, and so on.^17^ The above clinical trials were safe and well tolerated after transplantation. No patients experienced serious adverse reactions or dose‐limiting toxicity caused by MSC transplantation, and no adverse outcomes were observed.^15^, ^16^

So Yoon Ahn treated three premature infants with severe intraventricular hemorrhage (IVH) with low‐dose intraventricular transplantation of MSCs and six infants with high‐dose MSCs. The results showed that the transplantation was well tolerated and the cerebrospinal fluid (IL)‐6 levels increased after the transplantation. The initial CSF IL‐6 and tumor necrosis factor‐α levels were significantly correlated with the baseline ventricular index.^15^ V Bhati performed ipsilateral MCA arterial infusion of stem cells in 10 patients with acute stroke 8–15 days after stroke onset in the intervention group and no infusion was performed in the control group. It was found that eight patients in the intervention group showed a good clinical outcome, with a modified Rankin scale score of less than 2, and four patients in the control group achieved this outcome.^16^ The levels of CD34(+) progenitor cells in peripheral blood of 32 patients with primary ICH within 12 h after onset were detected at admission and 7 ± 1 day after the operation. It was found that the level of CD34(+) progenitor cells on Day 7 was an independent related factor for good prognosis at 3 months after the operation. These results suggest that CD34(+) progenitor cells may be involved in the functional recovery of patients with ICH.^18^

#### 1.2.2 Stem cell treatment in brain ischemia

**Figure 1:**
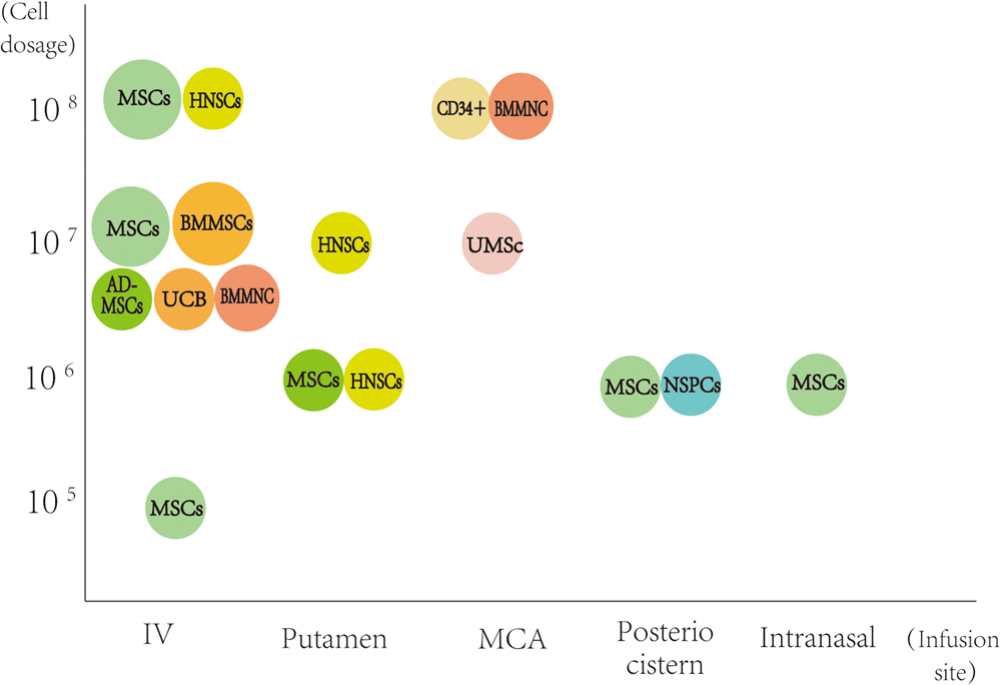
Visualization of the relationship between cell types, dose, and infusion site in clinical trials. The circled area represents the number of stem cell trials using the treatment. The bigger the circle, the more these experiments. AD‐MSCs, adipose‐derived mesenchymal stem cells; BMMSCs, bone marrow mesenchymal stem cells; HNSCs, human neural stem cells; IV, injection of vein; MCA, middle cerebral artery; MSCs, mesenchymal stem cells; NSPCs, neural stem/progenitor cells; UCB, umbilical cord blood; UMSc, umbilical cord mesenchymal stem cells.

There were 29 articles related to stem cell treatment in brain ischemia. The phase of clinical trials, cell interventions, dosage, infusion site, number of the trials (number of controls), main outcomes, safety, and year are listed as follows (Table 3, Figure 1).

**Table 3:** Summary of clinical trials of stem cells in the treatment of cerebral ischemia.

Cells	Dosage	Site	Number of trials (controls)	Phase	Main outcomes	Safety	Year
Allogeneic MSCs	4.5–5 × 10^6^	Nasal	10	/	No unexpected structural brain abnormalities (at the 3‐month follow‐up).	Safe	2022^19^
Allogeneic MSCs	3/6/9 × 10^7^	IV	15	I	Safety over 1 year.	Safe	2019^20^
Allogeneic MSCs	9 × 10^7^	IV	21	II	Significant improvements in all behavioral endpoints and an increase in the Barthel index (at the 12‐month follow‐up).	Safe	2019^20^
Autologous MSCs	Not mentioned	IV	31 (13)	Ⅲ	Motor function improved, and imaging showed better results.	Uncertain	2022^21^
Autologous MSCs	1.2 × 10^8^	IV	9 (8)	II	Significantly improved infarct volume from baseline in the treatment group was detected (at the 12‐month follow‐up).	Safe	2021^22^
Autologous MSCs	5 × 10^7^	IV	16 (36)	/	The modified Rankin Scale (mRS) score decreased. Clinical improvement was associated with serum stromal cell‐derived factor‐1 levels and degree of external subventricular involvement. No significant side effects were observed.	Safe (during 5‐year follow‐up)	2010^23^
Autologous MSCs	1 × 10^8^	IV	5 (25)	I/II	The Barthel Index and the modified Rankin score continued to improve within 1 year.	Safe	2005^24^
Autologous AD‐MSCs	6 × 10^7^	IV	4 (9)	IIa	No related serious adverse events or tumors occurred (during the 2‐year follow‐up).	Safe	2022^25^
Allogeneic HNSCs CTX0E03	2 × 10^7^	The putamen	23	II	Some improvement in motor function was found in patients with residual upper‐extremity movement at baseline.	Safe	2020^26^
Allogeneic MSCs/NSPCs	3 × 10^7^/3 × 10^8^/3.6 × 10^8^	IV/cerebellomedullary cistern	6	/	Neurological function, degree of disability, and activities of daily living improved.	Safe (during the 2‐year follow‐up).	2014^27^
Autologous BMMSCs	6 × 10^7^	IV	25	I	Partial function was recovered (the median 90‐day mRS ES decreased by 1 point).	Uncertain	2019^28^
Autologous BMMNC and CD34+ cells	5 × 10^8^	MCA	10 (10)	/	It improved clinical outcomes.	Safe	2018^16^
Autologous BMMSCs	2.875 × 10^7^	IV	60 (60)	II	There was no benefit concerning the therapeutic effect of stroke.	Safe	2014^29^
Allogeneic BMMNCs	8 × 10^7^	IV	11	I	Seven patients had good clinical prognoses.	Uncertain	2012^30^
Allogeneic OECs, NPCs, UCMSCs, SCs	1–5 × 10^6^	Intracranial parenchymal implantation, intrathecal implantation, and intravenous administration	10	/	The neurological function was improved (during 6–24 months of follow‐up) (the Barthel index score and the clinical neurological deficit scale score increased).	Safe	2013^17^
Autologous CD34+ cells	≤1 × 10^8^	MCA	5	I	All patients had improved clinical function scores (modified Rankin score and NIHSS score) and reduced lesion volume (at the 6‐month follow‐up).	Safe	2014^31^
Autologous UCB	1–5 × 10^7^	IV	23 (46)	I	13 of the 18 babies who received cell therapy were alive, and 19 of the 46 babies who also received cooling were alive (at the 1‐year follow‐up).	Uncertain	2014^32^
Allogeneic UCMSCs	2 × 10^7^	MCA	4	/	Muscle strength and modified Rankin scale scores improved in patients with ischemic stroke, but not in those with hemorrhagic stroke. No major unexpected events were observed (during the 6‐month follow‐up).	Uncertain	2012^33^
Allogeneic HNSC CTX0E03	1 × 10^8^/3 × 10^8^	IV	20 (11)	/	Sensorimotor neuroplasticity improved and motor function recovered (at the 2‐year follow‐up). The Barthel index, NIHSS, and the modified Rankin score had no significant effect, but motor NIHSS and the motor Fugl–Meyer score significantly improved.	Uncertain	2013^34^
Allogeneic Ctx‐dp (Developed from CTX0E03)	2 × 10^6^/5 × 10^6^/10 × 10^6^/20 × 10^6^	Shell membrane	11	I	Neurological improvements were observed.	Safe (at the 2‐year follow‐up).	2016^35^
Allogeneic aldehyde dehydrogenase‐Bright Stem Cells (ALD‐401)	No specific data	IV	60 (40)	II	The incidence of small lesions on MRI was higher in the treatment groups, but there was no difference in the primary efficacy endpoint.	Uncertain	2019^36^
Autologous G‐CSF and PBSCs	3–8 × 10^6^	Subcutaneous	15 (15)	II	Improvements in stroke scale (NIHSS, ESS, and EMS) and functional outcome (mRS) were evident, and imaging showed improvement (at the 12‐month follow‐up).	Uncertain	2014^37^
G‐CSF	600 μg	/	40 (20)	IIb	The change from baseline in MRI ischemic lesion volume tended to decrease.	Uncertain	2012^38^
G‐CSF	600 μg	Subcutaneous	24 (12)	IIa	Dependence did not differ between groups compared with controls, and functional outcomes did not differ across G‐CSF doses (at the 3‐month follow‐up).	Safe	2006^39^
G‐CSF	900 μg	Subcutaneous	10	/	Neurological improvement was more pronounced in the G‐CSF group, uptake in the surrounding area of the G‐CSF group was significantly improved, and metabolic activity was positively correlated with EMS scores (at the 1‐year follow‐up).	Uncertain	2006^40^
Allogeneic Dental Pulp Stem Cells	1 × 10^8^/3 × 10^8^	IV	42 (34)	/	Results were not released	/	2022^41^
Allogeneic MSCs	6 × 10^7^	Intrathecal	59 (59)	II	Results were not released	/	2019^42^
Autologous BMSCS	2 × 10^7^/5 × 10^7^	Around the Infarct area	6 ~ 10	I	Results were not released	/	2017^43^
Autologous Dental Pulp stem Cells	1 × 10^7^	Peri‐Infarct area	9	I	Results were not released	/	2016^44^
Autologous BMMSCs	1.2 × 10^8^/3 × 10^8^	Within the arteries	76	II	Results were not released	/	2015^45^

*Note*: Phase refers to the phase of clinical trials. Dosage in the header refers to the cell dose. Cell doses were re‐calculated at 60 kg for each patient if the dose was only stated in terms of the number of cells per kilogram (cells/kg). The judgment criteria of security is the patients were well‐tolerated, and no serious adverse events occurred during follow‐up.

Abbreviations: AD‐MSCs, amniotic‐derived mesenchymal stem cells; BMMSCs, bone marrow mesenchymal stem cells; G‐CSF, Granulocyte colony‐stimulating factor; HNSC, human neural stem cell; IV, intravenous; MCA, middle cerebral artery; MSCs, mesenchymal stem cells; NPCs, neural progenitor cells; NSPCs, neural stem/progenitor cells; OECs, olfactory ensheathing cells; PBSCs, peripheral blood stem cells; SCs, Schwann cells; UCB, umbilical cord blood; UCMSCs, umbilical cord mesenchymal stromal cells.

A large number of new clinical trials on cerebral ischemia have been conducted in recent years. In general, the momentum of stem cell therapy for cerebral ischemia into the clinic is strong. There are a variety of intervention methods, among which MSCs, BMMSCs, and NSCs account for the largest part of the cell intervention, followed by umbilical cord mesenchymal cells, CD34+ cells, olfactory ensheathing cells, Schwann cells, and dental pulp stem cells. Intervention methods also include the use of G‐CSF, which indirectly regulates the mobilization of stem cells. Overall, the safety of stem cells in the treatment of cerebral ischemia is satisfactory. The adverse events related to the intervention were mild or not significantly different from those in the control group, and no unexpected brain structural abnormalities were observed.^19^, ^25^ In addition to bone marrow‐derived stem cells (such as MSCs and BMMSCs) and CD34+ cells, which are commonly transplanted after autologous cell extraction, the rest of the cells are not easy to extract from themselves, and the treatment mostly involves allogeneic stem cell transplantation, which requires the use of immunosuppressive drugs. The good news, however, is that none of the clinical trials showed oncological or other serious adverse events during follow‐up.^26^ The number of cell injections varied, ranging from 5 × 10^5^ to 3 × 10^8^, and the number of injections was mostly one, with only four trials using multiple repeated cell treatments.^23^, ^27^, ^32^, ^42^ Multiple injections did not show significant safety changes compared to single injections, and patient symptoms also showed some improvement. The route of injection is also relatively diverse. MSCs are mostly injected intravenously, and in some trials, are injections were administered through the ipsilateral putamen of cerebral infarction or intranasally. For other cell types, the MCA route, intrathecal, cerebellomedullary cisterna, and intracerebral infarct area were mostly selected.^17^, ^20^, ^22^ The number of injected cells and the volume of solution around the cerebellomedullary cistern and infarct site are relatively small due to the smaller accommodating space than the arterioles and veins. For G‐CSF, the injection dose is the standard 10 μg/kg injection for 5 days by a subcutaneous injection.^39^, ^40^ The modified Rankin scale, the NIHSS scale, the mRS score, the Fugl‐Meyer assessment scale, the ARAT test, the European Stroke Scale (ESS), the ESS Motor Subscale (EMS), and the Barthel index (BI) were used to evaluate the function of cerebral ischemia before and after the intervention. Also, imaging and blood biochemical measurements of infarct size and blood biomarkers and other comprehensive assessments were performed.^22^, ^34^, ^40^

Acute cerebral ischemia is defined as cerebral ischemia within 24 h to 1 week after onset. Stem cell therapy for acute cerebral ischemia is safe, but the efficacy is variable. In a single‐arm, phase I clinical trial of bone marrow harvesting and intravenous infusion of autologous BMMNCs (10 million cells/kg) in patients with moderate acute ischemic stroke within 24–72 h after onset, treated patients showed a 1‐point reduction in ES in mRS at Day 90, with safe outcomes.^28^ In another prospective study, Banerjee et al. carried out a nonrandomized, open‐label, phase I study, which involved infusion of autologous CD34+ cells through the middle cerebral artery in five patients with severe anterior circulation ischemic stroke who presented within 7 days of symptom onset; improvement in clinical function scores and reduction in lesion volume were observed at 6 months of follow‐up in all patients.^31^ G‐CSF has also shown the potential to effectively mobilize bone marrow CD34+ stem cells in patients with recent ischemic stroke.^39^ However, some trials revealed that the treatment effect is not significant. Bone marrow‐derived allogeneic Baak LM et al. delivered bone marrow‐derived allogeneic MSCs intranasally for the treatment of 10 term neonates with perinatal arterial ischemic stroke; after 4 months, four infants showed asymmetric corticospinal tracts and three had abnormal early movements with no established treatment effect.^19^ Another phase II A, randomized, double‐blind, placebo‐controlled, single‐center pilot trial showed similarly modest efficacy.^25^ Four patients aged ≥60 years with moderate to severe stroke were treated with IV (intravenous) adipose tissue‐derived mesenchymal stem cells (Ad‐Mscs) within 2 weeks of onset. The median NIHSS score was lower at 24 months of follow‐up, but the difference was not significant. There were no differences in the mRS scores between the two groups.

Cerebral ischemia with a duration of 1–3 weeks after onset is called subacute cerebral ischemia. Stem cell therapy for subacute cerebral ischemia has shown reliable safety, and the efficacy is related to the individual differences of patients. A case of a test using IV injection of autologous bone marrow derived MSCs in the treatment of subacute moderately severe ischemic of carotid artery stroke test, a IV therapy using autologous BMMSCs within 7–30 days of the involvement of the patients with ischemic stroke and another case of former cycle ipsilateral MCA arterial infusion of stem cells in treatment of test results shows good feasibility of treatment, and improvement of motor neural plasticity.^16^, ^30^, ^34^ Otherwise, treatment with ipsilateral MCA intraarterial infusion of stem cells has also shown favorable clinical outcomes in the majority of patients. However, the efficacy of IV infusions has been questioned in some trials. Kameshwar Prasad conducted a phase II, multicenter, parallel‐group, randomized trial using an IV infusion of autologous BMSCs and found no benefit for stroke treatment.^30^ Another similar trial showed a significant improvement from baseline in terms of the absolute change in the median infarct volume but not in the total infarct volume in the treatment group. There were no statistically significant differences in the median NIHSS, mRS, or BI scores at 12 months compared with the control group.^22^ Savitz SI, using a carotid infusion of ALD‐401 in 100 patients with middle cerebral artery ischemic stroke, showed no difference in the primary efficacy endpoint and a higher incidence of small lesions on MRI in the treatment group compared with the control group.^36^ Notably, Huns001‐01 was injected directly around the subacute infarct area, suggesting that an intratumoral injection may be a more favorable method for delivering cells into the lesion site and improving motor function.^43^ In a phase II B, single‐center, randomized, controlled trial of G‐CSF treatment, Timothy J England found a trend toward reduced changes from baseline in MRI ischemic lesion volume, suggesting that subacute administration of G‐CSF is safe and that CD34(+) iron labeling in patients with ischemic stroke is feasible.^38^ Hideo Shichinohe conducted a phase I open‐label, uncontrolled dose–response study in which the bone marrow was harvested from each patient's iliac crest 15 days or later after symptom onset, treated as HunS001‐01, and injected around the subacute infarct area. The results suggest that intraparenchymal injections may be a more favorable approach for delivering cells to the lesion site and improving motor function.^43^ The current stage of stem cell therapy has a certain effect and can ensure safety, but the choice of intervention and the condition of the patients will greatly affect the outcome. More clinical trials are needed for comparison of intervention pathways, intervention cells, and the number of intervention cells to obtain more effective and exact effect data.

Treatment initiated 3 weeks after ischemic stroke was considered to be treatment in the chronic phase. At present, there is no effective treatment for chronic stroke. Fortunately, a considerable number of patients show significant improvement in behavioral endpoints and recovery of neurological and motor function after stem cell treatment.^17^, ^20^, ^37^ It is worth noting that using the allogeneic human neural stem cell line CTX0E03, Keith W Muir et al. performed implantation into the ipsilateral putamen of cerebral infarction in 23 adults with significant upper limb motor deficits 2–13 months after ischemic stroke; ARAT improvement was seen only in patients with residual upper limb movement at baseline.^26^ A single infusion of 2 × 10^7^ UCMscs via a catheter into the M1 segment of the middle cerebral artery by Yongjun Jiang improved muscle strength and modified Rankin scale scores in patients with ischemic stroke, but not in patients with hemorrhagic stroke.^33^ Jin Soo Lee conducted an open‐label, observer‐blinded clinical trial on 85 patients with severe cerebral artery regional infarction. Patients with ischemic stroke received an IV injection of autologous cultured MSCs, while the control group did not receive that individual quarantine. The degree of improvement was related to the level of serum stromal cell‐derived factor‐1 and the degree of external subventricular region involvement.^23^

### 1.3 Stem cell clinical trials for neurodegenerative disease

There are many neurodegenerative diseases, such as Huntington's chorea, spinocerebellar ataxia, motor neuron disease, and so on. Here, two diseases, AD and PD, are selected as representatives to discuss and summarize the current applications in stem cell clinical trials.

#### 1.3.1 Stem cell treatment in PD

There are nine valid articles related to stem cell treatment in PD. We read the articles and extract the phase of clinical trials, cell interventions, dosage, infusion site, number of the trials (number of controls), main outcomes, safety, and year as the header list below (Table 4).

**Table 4:** Summary of clinical trials of stem cell therapy for Parkinson's disease (PD).

Cells	Dosage	Site	Number of trials (controls)	Phase	Main outcomes	Safety	Year
Allogeneic NPC	2 × 10^6^	The putamen	8	/	Motor improvement and a trend of increased midbrain dopaminergic activity (at the 1‐year follow‐up). Improvement in UPDRS III in OFF conditions, and enhanced midbrain dopaminergic neurotransmission was observed (at 4 years of follow‐up).	Safe	2018^46^
Allogeneic NPC	3 × 10^7^	Unilateral striatum	21	/	The symptoms improved.	Safe	2016^47^
Autologous MSCs	1.02 × 10^8^	Intra‐arterial (intracranial)	5	I	The motor function rating scale remained stable for all six months.	Uncertain	2016^48^
Autologous BM‐MSCs	6 × 10^7^	Lateral ventricle	7	/	Three patients showed steady improvement on the Unified PD Rating Scale (UPDRS), and subjective improvements were found in symptoms. No serious adverse events occurred during 12–36 months after surgery.	Safe	2009^49^
Autologous SVF	6 × 10^7^	Facial muscles and nasal cavity	2	/	Qualitative improvements in postinjection motor and nonmotor symptoms, obvious reductions in PDQ‐39 and UPDRS motor scores (at the 1‐year and 5‐year follow‐up).	Uncertain	2020^50^
Allogeneic Embryonic dopamine cells	Not mentioned	The putamen	34 (6)	/	Significant improvement in symptoms (1–2 years of follow‐up).	Uncertain	2003^51^
Allogeneic Human embryonic dopamine‐neuron	Not mentioned	The putamen	20 (20)	/	Human embryonic dopamine‐neuron transplantation survives in patients with severe PD and yields some clinical benefit in younger patients (60 years old or younger), but not in older patients.	Uncertain	2001^52^
Allogeneic hRPE	1 × 10^6^	The postcommissural putamen	12	I/II	Improvement peaking of Scale‐M scores was at 12 months and then declined over the next 24 months. The surgery was well tolerated (at 12–36 months of follow‐up).	Safe	2012^53^
Autologous stem cells with superselective arterial catheterization	/	Intra‐arterial (intracranial)	50	/	A median 51.1% improvement on the Unified PD rating scale, and neurologic function improved in the majority of patients.	Safe	2010^54^
					No signs of tumor or stroke were found (at 4–9 months of follow‐up).		

*Note*: Phase refers to phase of clinical trials. Dosage in header refers to cell dose. Cell doses were re‐calculated at 60 kg for each patient if the dose was only stated in terms of the number of cells per kilogram (cells/kg). The judgment criteria of security is the patients were well‐tolerated, and no serious adverse events occurred during follow‐up.

Abbreviations: BM‐MSCs, bone marrow mesenchymal stem cells; hRPE, human retinal pigment epithelium; MSCs, mesenchymal stem cells; NPC, neural progenitor cell; SVF, stromal vascular fraction.

The most commonly used interventions are NPC, MSCs, and others including SVF, embryonic dopamine cells, hRPE, and stem cells with superselective arterial catheterization, among which MSCs, SVF, and stem cells with superselective arterial catheterization can be administered by autologous transplantation, while other cells are administered by allogeneic transplantation. The doses of cell grafts were relatively similar, ranging from a minimum of 1 × 10^6^ cells to a maximum of 3 × 10^7^ cells, most of which were concentrated in the order of 106. Most of them were single transplantations. Only one study used two unilateral striatal injections of 3 × 10^7^ NPC each at an interval of 7–57 months, which was the largest dose and the largest number of transplants in all stem cell therapies for PD disease, and the outcome was safe without significant side effects.^47^ The injection sites were the putamen, striatum, lateral ventricles, intra‐arterial (intracranial), facial muscles, and the nasal cavity. The scales used included PDQ‐39, UPDRS, Hoehn‐Yahr and Schwab‐England, PSP, Unified Parkinson's Disease Rating Scale, and QOL.^48^, ^50^, ^54^

The intervention can improve the neurological function of patients, improve the symptoms of PD, or maintain the stability of motor function. No serious adverse events or side effects were found in all the trials, and the safety was good, without signs of tumor or stroke.^46^, ^49^, ^54^ After transplantation of human neural progenitor cells, six of seven patients showed varying degrees of motor improvement and a trend of enhanced midbrain dopaminergic activity at the 1‐year follow‐up. At the 4‐year follow‐up, it was found that undifferentiated NPCS could be safely delivered to the putamen of PD patients using a stereotaxic approach, and midbrain dopaminergic neurotransmission was enhanced.^46^ Two other trials of MSCS transplantation were conducted, although one route was intra‐arterial and the other was intraventricular, and both showed significant improvements in symptoms such as facial expression, gait, and freezing episodes.^48^, ^49^ Autologous implantation of stem cells with superselective arterial catheterization in 50 patients showed a median 51.1% improvement on a unified PD rating scale, and eight patients showed an increased N‐acetylaspartate/creatine ratio in the right and left basal ganglia.^54^ One study found that human embryonic dopaminergic‐neuron transplantation survised in patients with severe PD and produced some clinical benefit in younger (<60 years) patients, but not in older (>60 years) patients, with fiber growth and increased 18 F‐fluorodopamine uptake in transplanted neurons from younger subjects. Yet, after the first year of improvement, 15% of the patients who received the transplant experienced recurrent dystonia and dyskinesia.^52^ Two years later, the authors conducted a second clinical trial of putaminal transfer of embryonic dopamine cells and showed that the symptoms of patients in the cell transplantation treatment group were significantly improved, but 4 out of 34 transplanters had secondary dyskinesia after significant clinical improvement within 1–2 years.^51^ We need to be alert to the relationship between this age cutoff and the benefit of treatment. In future trials, we should expand the samples and carefully explore other factors that affect the clinical efficacy to maximize the advantages and avoid the disadvantages, and benefit the public.

#### 1.3.2 AD and stem cell treatment

We screened and stayed nine valid articles related to stem cell treatment in AD. The phase of clinical trials, cell interventions, dosage, infusion site, number of the trial's cases (number of controls), main outcomes, safety, and year were extracted and are listed as follows (Table 5).

**Table 5:** Summary of clinical trials of stem cell therapy for AD.

Cells	Dosage	Site	Number of trials (controls)	Phase	Main outcomes	Safety	Year
Autologous hUCB‐MSCs	1 × 10^7^ (6)/3 × 10^7^(3)	Lateral ventricle	9	I/IIa	The most common adverse events were fever, headache, nausea, and vomiting, with three serious adverse events (Follow‐up for 3 months). No further serious adverse events occurred (follow‐up for 36 months).	Unsafe	2021^55^
G‐CSF	600 μg	Not mentioned	5(3)	IIa	Mean paired association learning improved significantly, with no significant difference in cerebrospinal fluid amyloid‐β1‐42 levels.	Safe	2012^56^

*Note*: Phase refers to the phase of clinical trials. Dosage in the header refers to the cell dose. Cell doses were re‐calculated at 60 kg for each patient if the dose was only stated in terms of the number of cells per kilogram (cells/kg). The judgment criteria of security is the patients were well‐tolerated, and no serious adverse events occurred during follow‐up. hUCB‐MSCs: human umbilical cord blood mesenchymal stem cells; and G‐CSF, granulocyte colony‐stimulating factor.

At present, the number of clinical trials of stem cell therapy for human AD is relatively small, and there is a large gap. Hee Jin Kim conducted a phase I clinical trial in nine patients with mild to moderate Alzheimer's dementia.^55^ Four weeks before administration, the Ommaya reservoir was implanted into the right lateral ventricle of the patients. Six patients received a high dose of hucB‐Mscs (3.0 × 107 cells/2 mL in all patients in triplicate injections, 4 weeks apart). Follow‐up for 3 months showed that there were three serious adverse events thought to be caused by the study products, and other adverse reactions such as fever and headache all resolved within 36 h. At a further 36 months of follow‐up, no further serious adverse events were observed. Juan Sanchez‐Ramos conducted a double‐blind, placebo‐controlled, crossover design to administer G‐CSF to eight patients with mild to moderate AD for 5 days. No serious adverse events were identified, the most common side effects being transient leukocyte increase, myalgia, and diffuse pain. The results showed that the average paired association learning improved significantly. There was no significant difference in cerebrospinal fluid amyloid‐β1–42 levels between 2 weeks of G‐CSF treatment and placebo treatment.^56^ In addition, Maler found that early Alzheimer's dementia was significantly associated with age, cerebrospinal fluid β‐amyloid (Abeta) 1–42, and the Abeta42/40 ratio, and the obtained data suggested that regenerative hematopoietic support in the central nervous system was insufficient in the early stage of AD.^57^

### 1.4 Problems to be considered in stem cell clinical trials

#### 1.4.1 Immunogenicity of stem cells

Stem cells used in clinical trials are mostly autologous and allogeneic, of which allogeneic stem cells are more commonly used because of their convenience and easy availability. However, stem cells are immunogenic and potentially trigger both adaptive and innate immunity. Most in vitro studies have highlighted the immunosuppressive properties of MSCs, but mismatched MSCs are still immunogenic. Although allogeneic MSCs does not elicit rapid rejection in immunocompetent hosts like mismatched fibroblasts or HSCs, rejection of allogeneic MSCs was slower than that of other allogeneic cell types. However, it is inappropriate to suggest that MSCs are immunologically privileged because they do elicit humoral and cellular immune responses in vivo. The timing and severity of rejection to MSC appear to depend largely on the context and on the balance between immunogenicity and immunosuppressive factor MSC expression. Mismatched allogeneic and allogeneic MSCs can be preferentially present in immunosuppressed in vivo environments, such as tumors, but cannot persist in other tissues within the same animal.^58^, ^59^ This suggested that local immunosuppression was required to mask MSC immunogenicity.

A key example of differential immunogenicity is not only HLA‐dependent allorecognition but also the differential expression of high procoagulant tissue factor (TF/CD142) between different cell products. Different immediate blood‐mediated inflammatory response (IBMIR) triggeres differently, which is a major safety concern for intravascular use of different stem cell products. Cells that are not in regular contact with blood often express different amounts of highly procoagulable tissue factor (TF/CD142), and if MSCs are considered to be derived from perivascular cells or pericytes, then TF/CD142 expression serves the biological function of stopping bleeding by triggering thrombosis in response to vascular injury.^60^, ^61^, ^62^ In patients who are not properly anticoagulated, potentially fatal adverse events such as thrombosis and embolism can result.^63^, ^64^ These reports of adverse events and cases highlight the clinical need for new criteria for blood compatibility assessment. MSCs derived from different tissues vary greatly in their blood compatibility.^65^, ^66^ Infusion of procoagulants expressing the cellular products of tfscs, such as mesenchymal stem cells, hepatocytes, and islets, triggers innate immune attack (IBMIR), which affects cell engraftment, safety, and efficacy. In general, AT‐ and PT‐derived MSC products express higher levels of TF/CD142, thereby triggering a stronger IBMIR with concomitant thromboembolic events, unless carefully antagonized with anticoagulants. Therefore, blood compatibility tests must be performed in clinical work, and CAT and TEG are commonly used in clinical practice to obtain adequate safety estimates.^67^, ^68^

#### 1.4.2 Cryopreservation and freeze–thawing

Cryopreservation and its associated freezing and thawing procedures are one of the final steps in the manufacture and clinical application of a variety of common and feasible cell therapies, but the differences in viability and function between frozen stem cells and fresh stem cells cannot be ignored. Although there are some preclinical data suggesting that thawed MSCs are somehow equivalent to fresh MSCs, there are also some studies confirming that fresh low‐passage MSCs can provide more effective therapy.^69^, ^70^, ^71^, ^72^ Cryopreservation below −135°C can effectively stop cell activity, but storage often encounters some warming events that can cause transient warmth, for example, due to frequent cell batches being removed/introduced from storage devices, which can affect or even damage any cells that remain in the cold environment for a long time. This is especially true when the temperature is increased repeatedly to the extent that it may affect/impair the cell mass. Alternatively, a major problem with cryopreservation of HSPCs is reduced viability due to cell damage during freezing and thawing.^73^ Such damage can be caused by osmotic disturbance, ice crystal formation, and cellular dehydration.^74^ Freezing and thawing, especially the formation of exothermic ice crystals, can puncture and thus damage cell membranes. Thawed cells readily recover from freezing and can show a higher degree of membrane asymmetry, phosphatidylserine exposure, heat shock protein expression, and actin–cytoskeleton disruption. These processes are associated with potential changes in osmolality, pH, and temperature and must be effectively controlled.^75^ In addition, frozen–thawed MSCs also elicited stronger innate and adaptive immune responses and were more likely to trigger an IBMIR.^76^ The complement activity human serum exposure assay showed that thawed MSCs undergo more rapid lysis due to increased complement activation, and that thawed MSCs may undergo more rapid lysis in vivo via IBMR‐mediated detrimental processes immediately after infusion.^71^ A recent study demonstrated better clinical outcomes with fresh and low passage cells, with patients treated with freshly harvested cells in low passage having response rates twice as high as those in the frozen–thawed cell treatment group, and impaired immunomodulatory and hemomodulatory properties of cryopreserved MSCs after thawing, leading to faster complement‐mediated elimination after blood exposure.^76^

It has been suggested that cryopreserved HSPCS should be infused as soon as possible after thawing, including freezing medium, to maintain their optimal cell viability and function, but prior washing/removal of cryoprotectants is also recommended in the literature, especially if the patient weighs less than 20 kg.^77^, ^78^ Although removal of DMSO may be ideal to prevent undesirable toxicity, DMSO elution carries a risk of cell graft contamination/damage and requires actual cell handling in the field. These factors raise the possibility of inducing apoptosis in the cell graft, which may delay HSPC engraftment in the patient.^73^ Techniques that minimize the reduction in survival of cryopreserved stem cells are being actively explored. One approach is to use poly‐D‐lysine (PDL)‐coated microgel encapsulation of MSC before cryopreservation, which needs to be robust enough, safe, and allow paracrine interactions and induction of immune regulation and regeneration.^79^ Similarly, incorporation of cells into hydrogels may be an effective way to extend/optimize their paracrine potency, which may also improve cell survival after thawing and in vivo application.^80^ Hornberger et al. summarized the techniques which lead to the greatest survival of stem cells after freezing, including alternative cryoprotectants and preinfusion cryoprotectants, and showed that the number of CD34+ cells after thawing was a good predictor of post‐infusion function.^73^ In conclusion, the study of freeze–thaw techniques may provide key advantages to improve the safety and efficacy of cell therapies.

## 2 SUMMARY AND PROSPECTS

The incidence rate of cerebral apoplexy and neurodegenerative diseases is gradually increasing in modern society, but there is no effective clinical treatment. A large number of basic research and animal experiments showed that regenerative medicine has great potential for use in current and future medical treatment. By using animal models, it is difficult to estimate which pathological aspects (time, degree, age, complications, etc.) of patients can benefit most from stem cell therapy. Therefore, clinical trials involving a large number of patients or real‐world data are needed to solve this problem. As far as the current clinical trials are concerned, almost all stem cell therapies for stroke and neurodegenerative diseases are safe, without tumor formation and related major adverse events. There are many experimental results of a stroke, especially cerebral ischemia, showing the effectiveness of its treatment (although the effectiveness of different trials varies greatly). The number of clinical trials of AD and PD is small, but they also show effectiveness and safety, suggesting that more stem cell clinical trials can be carried out in the future to actively seek potential effective stem cell therapy.

In clinical trials, the outcome evaluation needs to be fully improved to accurately monitor the results of clinical trials. There are many scales used as one of the outcome evaluation methods in clinical trials, but different scales have their advantages and disadvantages, so we should choose carefully. For example, in stroke diseases, mRS, NIHSS, and BI are commonly used, but the scale of mRS is too broad to detect small differences, and NIHSS is mainly used for acute evaluation of patients. Second, clinical trials have been conducted in a variety of ways, and there is no consensus on the most effective method, including the timing of transplantation, cell type, cell dose, and route of transplantation. Before carrying out a larger clinical trial, it is necessary to further study the inclusion criteria, dosage, and effective and unified evaluation methods in the future to maximize the usefulness of the results and determine the effectiveness of the results.

## AUTHOR CONTRIBUTIONS

Yong‐Xin Bao and Senio Campos de Souza contributed to the central idea. Shan‐Shan Yan completed the literature search, collation, and writing of the paper. Zhen‐Dong Xie and Shan‐Shan Yan revised the manuscript.

## CONFLICT OF INTEREST STATEMENT

The authors declare no conflict of interest.

## ETHICS STATEMENT

Not Applicable.

## Data Availability

The data that support the findings of this study are available from the corresponding author upon reasonable request.
